# Efficacy of Cognitive-Behavioral Therapy for the Prophylaxis of Migraine in Adults: A Three-Armed Randomized Controlled Trial

**DOI:** 10.3389/fneur.2022.852616

**Published:** 2022-04-28

**Authors:** Timo Klan, Charly Gaul, Eva Liesering-Latta, Bernhard Both, Isabella Held, Severin Hennemann, Michael Witthöft

**Affiliations:** ^1^Department of Psychology, Johannes Gutenberg University of Mainz, Mainz, Germany; ^2^Headache Center Frankfurt, Frankfurt, Germany; ^3^Migraine and Headache Clinic Königstein, Königstein, Germany

**Keywords:** migraine, prevention, randomized controlled trial, behavioral treatment, cognitive-behavioral therapy, relaxation therapy, behavioral therapy

## Abstract

**Background:**

Behavioral approaches are central to the preventive treatment of migraine but empirical evidence regarding efficacy and effectiveness is still sparse. This study aimed to evaluate the efficacy of a newly developed migraine-specific, integrative cognitive-behavioral therapy program (miCBT) combining several approaches (trigger and stress management, coping with fear of attacks, relaxation training) by comparing it with a single behavioral approach (relaxation training, RLX) as an active control group and a waiting-list control group (WLC).

**Methods:**

In a three-armed open-label randomized controlled trial, 121 adults with migraine were assigned to either miCBT, RLX or WLC. The outpatient group therapy (miCBT or RLX) consisted of seven sessions each 90 min. Participants who completed the WLC were subsequently randomized to one of the two treatment groups. Primary outcomes were headache days, headache-related disability, emotional distress, and self-efficacy. The baseline was compared to post-treatment, and followed by assessments 4- and 12-months post-treatment to compare miCBT and RLX.

**Results:**

Mixed-model analyses (intention-to-treat sample, 106 participants) showed significantly stronger pre-post improvements in self-efficacy (assessed by the Headache Management Self-Efficacy Scale, HMSE-G-SF) in both treatment groups compared to the WLC (mean difference at post; miCBT: 4.67 [0.55–8.78], *p* = 0.027; RLX: 4.42 [0.38 to 8.46], *p* = 0.032), whereas no other significant between-group differences were observed. The follow-up analyses revealed significant within-group improvements from baseline to 12-month follow-up in all four primary outcomes for both treatments. However, between-group effects (miCBT vs. RLX) were not significant at follow-up.

**Conclusion:**

The miCBT has no better treatment effects compared to RLX in migraine-prophylaxis. Both treatments effectively increase patients' self-efficacy.

**Trial Registration:**

German Clinical Trials Register (www.drks.de; DRKS-ID: DRKS00011111).

## Introduction

Migraine is a common, primary headache disorder, (1) and one of the leading causes of disability worldwide (2). The migraine pathogenesis is complex, and different neurophysiological processes or structures (e.g., hyperresponsivity of sensory cortices, trigeminovascular system) are involved (3). In addition, psychological factors (e.g., experience of stress, coping-style) are assumed to affect the course of the disease (4–6). Therapy consists of acute pain management and preventive strategies to reduce the frequency and intensity of migraine attacks. Preventive interventions in migraine comprise pharmacological treatments as well as behavioral treatments with a range of different approaches. Behavioral treatments for migraine prophylaxis are recommended as a fundamental intervention for all migraine patients. Pharmacological strategies should be added if needed (7). The different behavioral approaches for the preventive treatment of migraine can be classified into (i) patient education; (ii) relaxation training (RLX); (iii) biofeedback; (iv) cognitive-behavioral therapy (CBT); (v) mindfulness-based interventions, (7–9) and (vi) trigger management for primary headaches by “learning to cope with triggers” (LCT), which explicitly includes exposure-based strategies (10). Since different behavioral interventions are immanent in several behavioral approaches (e.g., LCT includes elements of CBT and relaxation), this classification serves more as a rough orientation rather than a clear distinction. CBT is primarily used to improve stress management (8, 9). However, in the last decade more migraine-specific CBT interventions emerged (e.g., coping with fear of attacks or addressing comorbid disorders, such as insomnia) (11, 12).

Several reviews provide evidence for the efficacy of behavioral approaches in migraine prophylaxis (13, 14). Relaxation, biofeedback, and CBT are presumably effective to a similar extent (14, 15). A meta-analysis on the efficacy of biofeedback for migraine prevention yielded a medium effect size (16). Different relaxation techniques are considered to have similar effects, with the advantage of Progressive Muscle Relaxation (PMR) being ease of learning and application (15). It has been shown that the regular use of PMR even has a positive impact on migraine-relevant physiological parameters (i.e., the initial Contingent Negative Variation, iCNV) (17). Already migraine-specific patient education and lifestyle counseling can lead to a reduction in attack frequency (15, 18). A recent randomized-controlled trial showed that migraine-specific patient education and a mindfulness-based approach (i.e., mindfulness meditation) led to similar decreases in headache activity (19). The exposure-based LCT tended to have higher effects than other behavioral interventions, but some of these differences were marginal (20). Overall, no clear superiority in migraine prophylaxis can be attested to any of the behavioral approaches to date. Kropp and colleagues note that several reviews point to methodological limitations of previous studies on behavioral migraine prophylaxis (15). A recent Cochrane review concluded that evidence is still weak or of very low methodological quality, and that further studies are warranted (21). Another recent review found small to medium effects of psychological interventions on migraine prophylaxis and confirmed the need for further studies (22). One criticism of most previous behavioral approaches is that migraine-specific characteristics (e.g., recurring attacks of headache) or the management of triggers are not addressed sufficiently. Further, each behavioral approach in isolation might not cover the whole range of helpful interventions for behavioral migraine-prophylaxis, because specific pathological factors of patients (e.g., high trigger avoidance) are not treated adequately. To close this gap, different approaches were combined into a migraine-specific, integrative CBT program (miCBT) which demonstrated good feasibility in a single group pilot study (23).

CBT has a focus on changing dysfunctional, disease-related thoughts and beliefs. Thus, CBT is intended to reduce inappropriate, negative emotions and to establish a better coping with the disease. In the present miCBT, the exposure-based LCT and behavioral interventions, such as relaxation training, are integrated, so that this approach represents an extension compared to CBT programs that focus on modifying dysfunctional cognitions. This study aimed to assess the efficacy of the miCBT compared with a standard behavioral intervention (RLX) for migraine-prophylaxis and a waiting-list control group (WLC) in a three-armed randomized controlled trial. The long-term efficacy was evaluated by the comparison of miCBT with RLX in a 4- and 12-month follow-up assessment. It was hypothesized, that both behavioral interventions are superior to the WLC condition and that the miCBT leads to higher treatment effects compared to the RLX program.

## Methods

### Study Design and Participants

The study was designed as an open-label randomized controlled trial with two stages. The first stage (pre-post) comprised three conditions (miCBT, RLX, WLC), and the second stage (4- and 12-month follow-up) comprised two conditions (miCBT, RLX) since the pre-post completers of the WLC were randomly assigned to the miCBT- or RLX-condition. The study was conducted at the psychotherapy outpatient clinic of the Department of Psychology (University of Mainz, Germany). The study protocol was approved by the Ethics Committee of the State Chamber of Medicine in Rhineland-Palatinate, Germany, reference number 837.291.16 (10610).

Participants were recruited *via* flyers, social media, several local newspaper articles, and one TV clip. Inclusion criteria were (i) meeting the ICHD-3 beta criteria (24) of either migraine without aura, migraine with aura, or chronic migraine for at least one year; (ii) a minimum of four headache days per month, and a pattern of migraine symptoms stable over the last six months; (iii) psychological factors, such as dysfunctional thoughts (e.g., overambitious achievement orientation), emotions (e.g., attack-related fear), and behavior (e.g., excessive avoidance of triggers), or the experience of emotional distress, were associated with migraine [meeting the DSM-5 criteria (25) of either “somatic symptom disorder” or “psychological factors affecting other medical conditions”]; (iv) fluency in German, Internet access; (v) age of at least 18 years. Exclusion criteria were (i) diagnosis of medication-overuse headache; (ii) currently taking a headache prophylactic medication (3-month wash-out) or therapy with botulinum toxin or neuromodulation during the trial period; (iii) previous completed or current psychotherapy; (iv) a severe mental disorder or medical comorbidity (which was likely to interfere with the ability to participate in group therapy, e.g., an acute psychosis, a major depressive episode, or an advanced Parkinson's disease); (v) suicidal tendency; and (vi) pregnancy or lactating.

Pre-screening was performed by phone followed by a face-to-face screening session. The face-to-face session included a structured interview to validate the migraine- and DSM-5-diagnosis. When checking possible DSM-5 diagnoses, the individual criteria of the disorder were queried in each case if the answer to the corresponding screening question was positive. All participants had to give written informed consent. Since all screenings were conducted by Master's level clinical psychologists, the participants had to provide a medical certificate stating the migraine diagnosis.

### Randomization and Masking

Eligible participants were randomly assigned with a 1:1:1 ratio to miCBT, RLX, or the WLC using block-randomization. In a second stage, the completers of the WLC were randomly assigned to miCBT or RLX (1:1 ratio). Randomization was performed by an independent statistician, using a computer-generated random sequence. Participants were then informed by the principal investigator *via* email about their allocation (i.e., whether they were receiving a behavioral intervention or had been placed on the WLC). The exact type of intervention (miCBT or RLX) was disclosed in the first treatment session since the treatment programs provided that the participants were given an agenda, and since participants were assumed to realize the type of intervention in the course of the therapy anyway. Since all outcomes were self-reported data, blinding of outcome assessment was not provided.

### Interventions

Both treatments (miCBT, RLX) comprised seven, weekly group sessions, each lasting 90 min. The treatments were conducted by five Master's degree clinical psychologists. To avoid bias, each therapist provided a similar amount of both miCBT- and RLX-treatments. Therapists were trained in a 1-day workshop before conducting the treatment and received regular supervision at least two times per treatment cycle by the first author. Both interventions are described in detail in a treatment manual. Since a participation rate of at least five sessions was aimed for in both treatments, missed group sessions were made up in extra group or one-to-one sessions.

**Figure 1:**
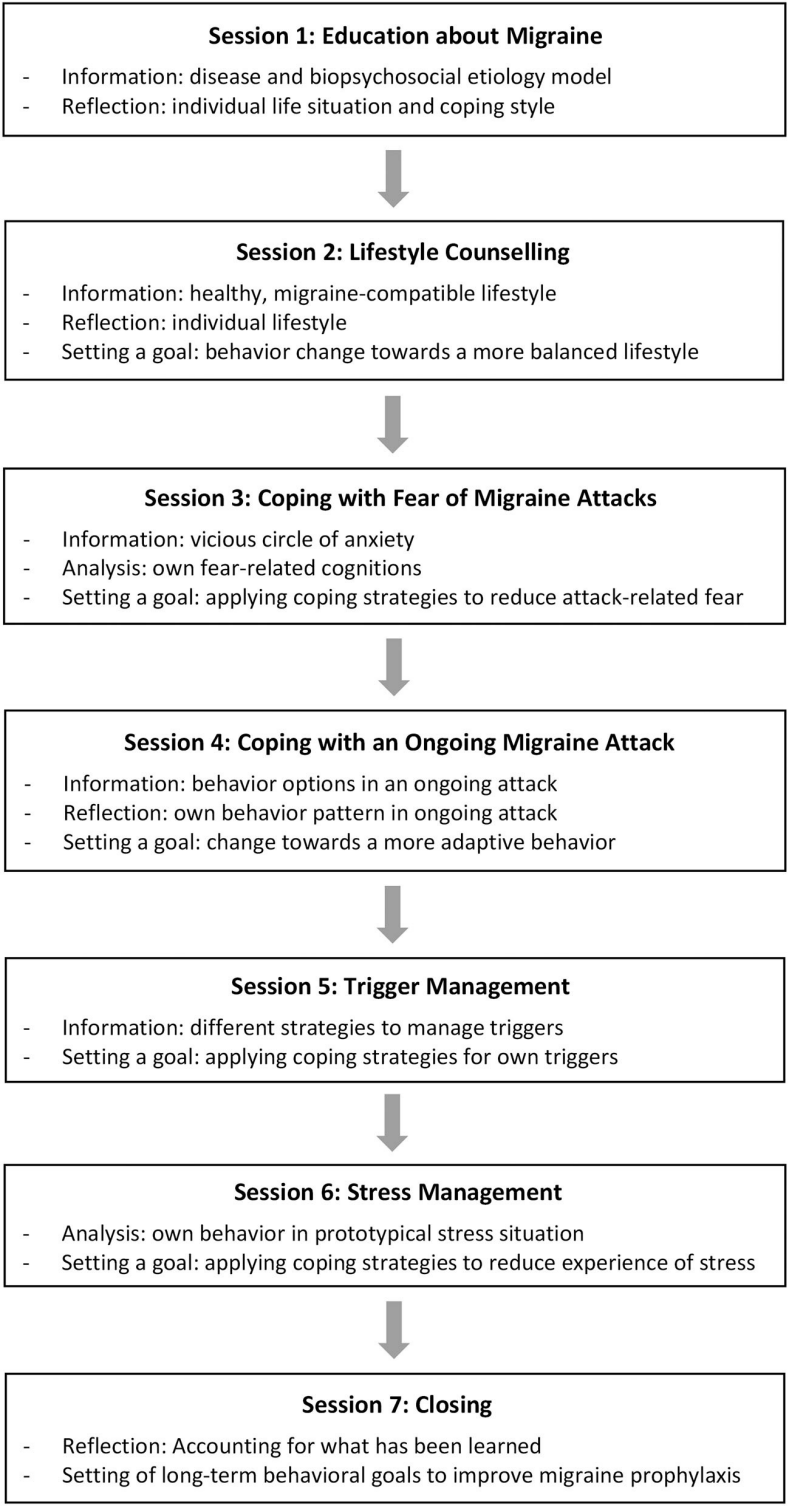
Migraine-specific, integrative cognitive-behavioral therapy program (miCBT).

The miCBT included a mixture of several behavioral approaches (Figure 1) (23). In session 1 (*Education about Migraine*) participants were informed about the disease (e.g., symptoms, different phases of a migraine attack) and the different factors, which are assumed to contribute to the emergence and maintenance of migraine or migraine attacks. A biopsychosocial etiology model of migraine, integrating pathophysiological findings (e.g., central neuronal hyperexcitability as an underlying vulnerability), psychological factors (e.g., dealing with triggers, especially stressors), and a threshold for migraine attacks was provided. Participants were encouraged to reflect on their own life situation and own coping styles regarding the etiology model. In session 2 (*Lifestyle Counseling*), basic recommendations to practice a healthy, migraine-compatible way of life (e.g., relaxation, recreation, and physical activity on a regular basis) were presented. The participants were motivated to formulate their own goals with regard to an even more balanced lifestyle. Session 3 (*Coping with Fear of Migraine Attacks*) contained behavioral techniques to analyze and modify fear-related cognitions. Thus, the participants were informed about the vicious circle of anxiety and how to cope with attack-related fear. In Session 4 (*Coping with an Ongoing Migraine Attack*) various behavioral options in managing an acute attack were discussed, with the assumption, that improved coping skills lead to decreased fear of attacks. In doing so, the four different issues (i) medication intake behavior, (ii) activity level, (iii) communication of attack-related complaints to other persons, and (iv) automatic thoughts were addressed. Above all, the participants were encouraged to reduce their activity level during an ongoing attack as far as possible. Subsequently, the participants were asked to reflect on their own typical behavior patterns in an ongoing attack and to formulate appropriate change goals in this regard. Session 5 (*Trigger Management*) provided five different strategies to manage triggers: (i) experiment, (ii) avoid, (iii) coping with stress, (iv) exposure, and (v) acceptance. The participants were asked to select suitable strategies for their own five most relevant triggers and to apply them in the subsequent. Session 6 (*Stress Management*) was focused on cognitive-behavioral techniques to improve coping with stress since emotional distress is regarded as the most important trigger in migraine attacks (6). Thus, the model of behavioral analysis was applied for the assessment of an individual, prototypical stressful situation (26). Subsequently, each participant was encouraged to consider a suitable coping strategy for this situation in the future. In Session 7 (*Closing*) the participants were encouraged to reflect on what they have learned and to formulate long-term behavioral goals for coping with their migraine. Each session (except for the first session) started with a reflection on the previous session (including feedback about successful or failed behavioral changes). Subsequently (except for the last session), the new issue (e.g., stress management) was introduced (including a referred behavioral analysis) and an individual formulation of behavioral change goals was encouraged. At the end of each session, the participants were taught a different, brief relaxation method.

The relaxation training (RLX) was aimed at teaching progressive muscle relaxation (PMR) and transferring it to everyday life. PMR is a systematic relaxation technique with Grade A in migraine prophylaxis (9). The RLX was developed based on the manual by Bernstein and Borkovec (27). In session 1, an education about migraine and PMR was provided. Further, the long PMR-version (16 muscle groups) was conducted. In session 2, the long PMR-version was conducted again, and a rationale for regular, independent practice was given. It was recommended to perform one relaxation exercise (lasting about 20 min) per day (15). In the following sessions 3–7, variations of PMR (version with 7 or 4 muscle groups, envision exercise, conditioned and differential relaxation) were taught. The participants were repeatedly encouraged to regularly perform PMR on their own. To support transfer into everyday life, participants were given logs to document their weekly exercise practice. Keeping the logs was not mandatory, but was strongly recommended. From the third session onward, the everyday life exercise practice was reported by each participant at the beginning of the session. Difficulties in implementation were addressed and appropriate ways to establish a regular exercise practice were developed.

### Assessments

All outcomes were assessed at baseline (pre-treatment), post-treatment, 4-month follow-up, and 12-month follow-up. Each assessment included a 4-week online diary and a one-time online survey (about 60 min). The online diaries were completed by recording hourly ratings of headache intensity using a 4-point rating scale from 0 (no headache) to 3 (severe). Additionally, the amount of daily pain medication had to be entered. Participants were instructed to enter data at least once per day. The frequency of data entry was monitored using specific software. If participants did not enter data for more than three days, they were reminded *via* a personal email or phone call. The online survey was conducted *via* SoSci-Survey (28) and contained a set of questionnaires on headache-related factors (e.g., disability). After the successful completion of the pre-measurement, the treatment (miCBT or RLX) or the waiting period (WLC) started. Immediately after the last treatment session (miCBT or RLX) or after seven weeks (WLC), the post-assessment was carried out. The WLC-participants were randomized to one of the treatments after the successful completion of the post-assessment. Follow-up assessments were conducted 4 and 12 months after the successful completion of the treatment. At the 12-month follow-up, participants were additionally asked, whether relaxation had continued to be practiced and to what extent.

After each treatment session, participants filled out the Group Therapy Session Evaluation by Patients (GTS-P) (29) as a paper-pencil questionnaire. The GTS-P comprises 8 items regarding the therapeutic process, including one item addressing treatment expectancy. Since there are no standardized methods for assessing the adverse effects of behavioral therapy given to adults with migraine, a free response field was added to each paper-pencil questionnaire. In doing so, the participants were given the opportunity to report adverse events as well as to make suggestions for changes to the sessions.

### Treatment Integrity

All therapy sessions (in total 112, excluding catch-up sessions) were videotaped. In each cycle, 2 sessions were randomly selected for evaluation (in total 32 sessions). The rating was done each by two Master's level clinical psychologists. Raters received training provided by the first author on how to apply the evaluation forms or scales. *Therapists' adherence* was rated on a 10-item evaluation form with a 3-point rating scale, with the analogous rating possibilities 0 = not adherent, 1 = partly adherent, and 2 = adherent. The evaluation forms were largely similar for both conditions. Both evaluation forms were developed following the Cognitive-Behavioral Maintenance Adherence Scale (30). *Therapists' competence* was assessed each with the German Version of the Cognitive Therapy Scale (adapted for group therapy, CTS-D-G) (31). The CTS-D-G comprises 18 items with each a 7-point rating scale from analogous 0 = poor to 6 = excellent.

### Outcomes

Four primary outcomes were defined: (i) headache days/28 days, (ii) headache-related disability, measured with the Headache Disability Inventory (HDI), (32) (iii) emotional distress, measured with the Depression Anxiety Stress Scales (DASS), (33) and (iv) headache-specific self-efficacy, measured with the German short form of the Headache Management Self-efficacy Scale (HMSE-G-SF) (34). The HDI assesses the impact of headaches on daily living. It includes 25 items and has a very good internal consistency (Cronbach's α = 0.90) (32). The DASS comprises three scales (Depression, DASS-D, Anxiety, DASS-A, and Stress, DASS-S). Each scale includes seven items (short version). The three scales have satisfying to good internal consistency (Cronbach's α = 0.78 to 0.92) (33). The HMSE-G-SF provides an assessment of headache-specific self-efficacy beliefs. It includes six items and has a satisfying internal consistency (Cronbach's α = 0.72) (34).

Secondary outcomes were (v) headache index/28 days, (vi) medication days, defined as the number of days of using headache medication for a 28-day period, (vii) sensitivity to and avoidance of headache triggers, measured with the Headache Triggers Sensitivity and Avoidance Questionnaire (HTSAQ), (35) (viii) headache-related disability, measured with the Pain Disability Index (PDI), (36) (ix) headache impact, measured with the Headache Impact Test (HIT-6), (37) and (x) acceptance of chronic pain, measured with the Chronic Pain Acceptance Questionnaire (CPAQ) (38). The HTSAQ requires an assessment of 26 potential triggers (24 common triggers and 2 triggers not listed before) using four scales: (i) *Triggers*, which refers to whether the named trigger is a trigger for the respondent's headaches, (ii) *Sensitivity to triggers compared with others, S (O)*, which refers to how sensitive the respondent is to the trigger compared with other persons, (iii) *Sensitivity to triggers compared with time of least sensitivity, S (T)*, which refers to how sensitive the respondent is to the trigger compared with the time of least sensitivity, and (iv) *Avoidance*, which refers to how hard the respondent tries to avoid the trigger. All four scales have a good internal consistency (Cronbach's α = 0.83 to 0.86) (39). The PDI includes seven items, each referring to disability in a specific area of life (e.g., social activity). Since the PDI assesses pain-related disability in general, the instruction was adapted toward headache-related disability. Its internal consistency is good (Cronbach's α = 0.85 to 0.90) (36). The HIT-6 includes six items and has a high internal consistency (Cronbach's α = 0.83) (40). The CPAQ includes 20 items and showed good internal consistency in a sample of persons with headache (Cronbach's α = 0.84) (39). All questionnaires were applied in a German Version.

**Figure 2:**
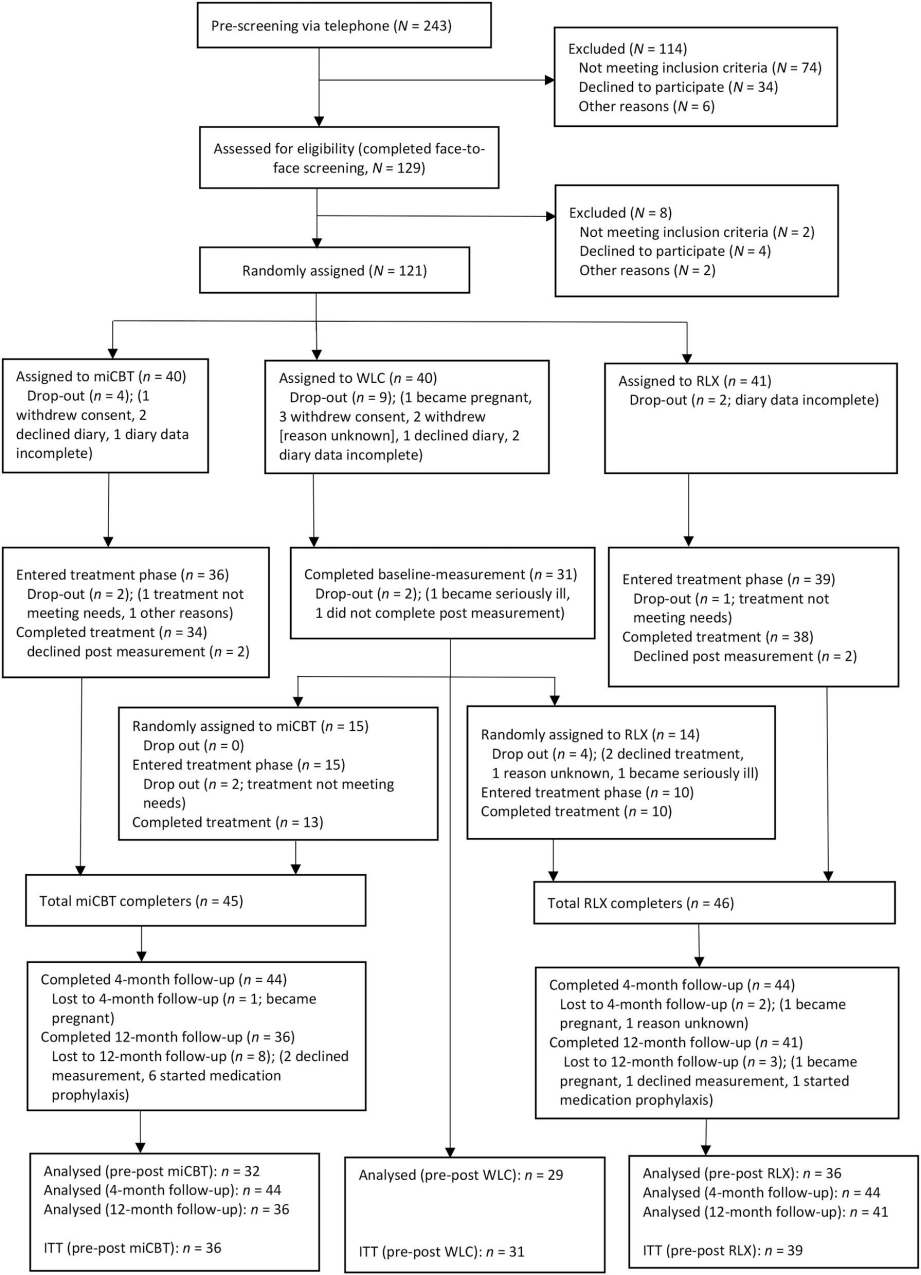
Participant flow. miCBT, migraine-specific, integrative cognitive-behavioral therapy program; RLX, relaxation training; WLC, waiting-list control-group; ITT, intention-to-treat.

A headache day was defined as “a day with headache pain that lasts ≥ 4 h with a peak severity of moderate or severe intensity, or of any severity or duration if the subject takes and responds to a triptan or ergot” (p. 490) or another headache medication (41). Since clinical experience shows that persons with migraine cannot always clearly assign existing headaches to migraine headache, it was decided to record headache days instead of migraine days. The headache index was determined by averaging the headache intensity ratings across the total day (24 h) and calculating a mean over the 28 days. Since the frequency of headache days per 28 days is more comprehensible than the headache index, this measure was defined as the primary outcome. To be analyzed, participants had to complete at least 80% of the diary entries (Figure 2) (42).

### Statistical Analyses

An a priori power analysis was conducted using G^*^Power (43). Based on previous evidence a moderate effect of miCBT vs. WLC was assumed (9). Thus, with Cohen's *f* = 0.25 at a power of 1 – β = 0.95 with an α = 0.05, a necessary sample size of at least 90 participants was calculated. Considering a dropout rate of 25%, the target sample size was increased to 120 participants.

Analyses were based on the intention-to-treat (ITT) sample, including all participants that were allocated to one of the three conditions and provided baseline data. To compare the change in outcomes from pre- to post-assessment between groups, mixed-model analyses for repeated measures (MMRMs) were conducted. Analyses included group (miCBT, RLX, or WLC), time (pre- and post-assessment), and the group × time interaction as fixed factors, as well as a random intercept (subjects) to model interindividual differences, with diagonal covariance matrices and using restricted maximum likelihood estimation. To analyze the second stage of this trial (long-term effects of miCBT vs. RLX), MMRMs were conducted with all participants that were allocated to one of the two treatments and provided baseline data (Figure 2). MMRMs included group (miCBT or RLX), time (pre, 4-month follow-up, 12-month follow-up), and the group × time interaction as fixed factors, as well as a random intercept to model interindividual differences, based on an auto-regressive structure with heterogenous variances (ARH1). Additionally, a sensitivity analysis using MMRMs was performed with complete cases (participants that completed ≥5 treatment sessions or WLC and provided data both at baseline and post-assessment). Since the analysis of covariance (ANCOVA) can be considered as robust method with high statistical power, (44) a further sensitivity analysis using this approach was performed. In view of the tightly calculated sample size, assuming medium-sized effects, a more permissive composite analysis was carried out *post-hoc*. The participants, which had received a behavioral treatment (either miCBT or RLX) between pre- and post-assessment were combined into one condition (“behavioral therapy”, BT) and compared with the participants of the WLC. Accordingly, an ANCOVA was performed with the outcomes' pre-values as a covariate and the outcomes' post-values as the dependent variable.

Within-group effect sizes were calculated as Cohen's *d*_av_ (45) (pre to 12-month follow-up) based on observed values. Responder analyses were performed for both follow-up assessments (4-month and 12-month follow-up). For the symptom-related outcomes (headache days, headache index, medication days), a reduction of ≥30% was defined as a response. In contrast to a more conservative ≥50% response operationalization, this threshold seems realistic to detect minor changes that can represent a veritable improvement for patients as well and can be considered more suitable for non-pharmacological interventions (46). For the psychometric questionnaires (e.g., HDI), the Reliable Change Index (RCI) (47) was calculated with a CI of 95% for observed values. For the 12-month follow-up, differences between the two treatment groups in terms of frequency and extent of relaxation practice were calculated using Fisher's exact test or a t-test. All analyses were performed with a two-tailed α of 0.05 in SPSS statistics version 23. A two-sided α was chosen to cover potential inferiority of the miCBT. This corresponds to the conservative approach commonly used in the scientific community (48, 49). We did not adjust the level of significance for multiple testing of primary outcomes to be able to control the type II error rate, to account for the pilot-character of this study in testing a newly developed miCBT, and since we compared distinct treatments with a WL control (50).

## Results

### Participants

Inclusion occurred between 9 January 2017 and 9 April 2019. In total, 243 persons were pre-screened by phone, which led to a face-to-face screening in 129 potential participants (Figure 2). Of these, 121 participants were randomly assigned to miCBT (40), RLX (41), or WLC (40), and 106 participants entered the intervention phase with a pre-measure (ITT; Table 1). Ninety-seven participants (91.5%) completed the post-measurement, and dropout rates were comparable between groups. Twenty-nine participants of the WLC thereafter were randomized to either miCBT (15) or RLX (14).

**Table 1:** Baseline characteristics (intention-to-treat sample, *N* = 106).

	**miCBT *n* = 36**	**RLX *n* = 39**	**WLC *n* = 31**
Age (years)	47.0 (11.3)	46.8 (13.9)	46.0 (10.7)
Disease duration (years)	22.2 (14.0)	21.8 (12.2)	21.9 (12.0)
Female	32 (89%)	34 (87%)	29 (94%)
In stable partnership	33 (92%)	31 (79%)	28 (90%)
Advanced level or degree after high school	26 (72%)	26 (67%)	21 (68%)
Employed	32 (89%)	28 (72%)	26 (84%)
Headache diagnosis			
Migraine without aura	30 (83%)	35 (90%)	24 (77%)
Migraine with aura	4 (11%)	3 (8%)	6 (19%)
Chronic migraine	2 (6%)	1 (3%)	1 (3%)
Mental disorder as comorbidity^a^			
No comorbid mental disorder	27 (75%)	26 (67%)	25 (81%)
Tentative diagnosis	4 (11%)	1 (3%)	1 (3%)
Comorbid mental disorder	5 (14%)	12 (31%)	5 (16%)
Previous experience in relaxation techniques	28 (78%)	23 (59%)	19 (61%)
Headache days per month (28 days)	9.0 (5.1)	8.6 (4.5)	7.4 (3.1)
Range	3 to 25	1 to 22	1 to 16
Headache index	0.28 (0.22)	0.27 (0.23)	0.21 (0.12)
Range	0.06 to 0.98	0.02 to 1.09	0.03 to 0.56
Medication days per month (28 days)	6.0 (4.1)	6.2 (3.9)	5.5 (3.1)
Range	0 to 18	0 to 20	0 to 11

*Data are n (%) or mean (standard deviation) unless otherwise stated. miCBT, migraine-specific, integrative cognitive-behavioral therapy program; RLX, relaxation training; WLC, waiting-list control-group.*

a *Presence of at least one mental disorder in addition to the DSM-5 diagnoses “somatic symptom disorder” or “psychological factors affecting other medical conditions”*.

The treatment sessions were conducted from 5 May 2017 to 18 July 2019. Each of the two programs (miCBT and RLX) was carried out eight times. Treatment sessions were conducted in groups of four to nine participants. The average session completion rate of the 91 treatment-completers (Figure 2) was high (miCBT: 93%, *M* = 6.53; SD = 0.59; range 5 to 7 sessions; RLX: 91%, *M* = 6.37; SD = 0.71; range 5–7 sessions).

### Treatment Integrity

*Therapists' adherence* was very high in both treatment conditions (miCBT: *M* = 1.80; SD = 0.52; RLX: *M* = 1.83; SD = 0.48). The inter-rater reliability was very high for each of the two evaluation forms, ICC (2,2) = 0.957, *p* < 0.001 (miCBT), ICC (2,2) = 0.980, *p* < 0.001 (RLX). *Therapists' competence* was also rated very high in both treatment conditions (miCBT: *M* = 5.24; SD = 0.98; RLX: *M* = 5.19; SD = 1.02). The inter-rater reliability was moderate for the CTS-D-G, ICC (2,2) = 0.571, *p* < 0.001. Thus, the treatment integrity was given in that a very high therapists' adherence as well as a very high therapists' competence (both assessments done with a statistically significant inter-rater reliability) were observed.

### Efficacy

ITT-analyses of the primary outcomes yielded significantly stronger pre-post improvements in self-efficacy (assessed by the HMSE-G-SF) in both treatments (miCBT: *p* = 0.027; RLX: *p* = 0.032) compared to the WLC, whereas no significant between-group differences were found for headache days, disability, and emotional distress (Table 2). The between-group comparisons for the secondary outcomes did not yield any statistically significant differences (Table 2, all *p*-values > 0.05). Sensitivity analyses with complete cases (*N* = 97) showed a similar pattern, except for a significant difference at post-assessment between RLX and WLC for the secondary outcome headache index in the ANCOVA (Supplementary Tables 1, 2; observed values in Supplementary Table 3). The *post-hoc* composite analysis by ANCOVA with complete cases yielded a significantly stronger pre-post improvement in self-efficacy as well as a significantly higher reduction in the headache index in the treatment condition (BT) compared to the WLC (Supplementary Table 4).

**Table 2:** Primary and secondary efficacy outcomes (by intention-to-treat, *N* = 106) of the pre-post analyses.

	**Within-group differences**						**Between-group differences**		
	**(Change from pre to post)**						**Mean difference at post (SE; 95% CI);** ***p-*****value**		
	**miCBT (*n* = 36)**		**RLX (*n* = 39)**		**WLC (*n* = 31)**		**miCBT vs. WLC**	**RLX vs. WLC**	**miCBT vs. RLX**
	**EMM**	**(SE)**	**EMM**	**(SE)**	**EMM**	**(SE)**			
**Primary outcomes**									
Headache days	−0.72	(0.66)	−1.47	(0.63)	0.33	(0.70)	0.59 (1.16; −1.72 to 2.90); 0.612	−0.57 (1.14; −2.82 to 1.68); 0.617	1.16 (1.10; −1.02 to 3.34); 0.294
Disability by HDI^a^	−5.01	(1.70)	−2.51	(1.63)	−6.03	(1.85)	1.87 (4.75; −7.55 to 11.29); 0.695	5.47 (4.66; −3.78 to 14.72); 0.244	−3.60 (4.48; −12.48 to 5.28); 0.423
Emotional distress (DASS)^a^	−2.83	(1.33)	−2.22	(1.28)	−0.43	(1.44)	2.80 (2.57; −2.30 to 7.90); 0.279	2.66 (2.52; −2.34 to 7.67); 0.294	0.13 (2.42; −4.67 to 4.94); 0.956
Self-efficacy (HMSE-G-SF)^b^	4.97	(1.52)	7.57	(1.46)	2.50	(1.64)	4.67 (2.07; 0.55 to 8.78); 0.027	4.42 (2.04; 0.38 to 8.46); 0.032	0.25 (1.95; −3.62 to 4.12); 0.899
**Secondary outcomes**									
Headache index	−0.03	(0.02)	−0.07	(0.02)	0.02	(0.02)	0.02 (0.05; −0.07 to 0.11); 0.664	−0.03 (0.05; −0.12 to 0.06); 0.486	0.05 (0.04; −0.04 to 0.14); 0.239
Medication days	−0.67	(0.62)	−0.70	(0.58)	0.45	(0.65)	0.01 (1.03; −2.04 to 2.07); 0.990	−0.42 (1.01; −2.43 to 1.58); 0.676	0.44 (0.98; −1.51 to 2.38); 0.656
Triggers (HTSAQ)^a^									
Scale triggers	1.75	(1.24)	0.26	(1.19)	−0.29	(1.34)	2.02 (3.04; −4.00 to 8.04); 0.508	0.31 (2.98; −5.60 to 6.22); 0.918	1.71 (2.86; −3.96 to 7.38); 0.551
Scale S (O)	1.60	(1.48)	0.26	(1.41)	−0.18	(1.60)	1.57 (3.61; −5.59 to 8.73) 0.665	1.39 (3.55; −5.64 to 8.42); 0.696	0.18 (3.40; −6.57 to 6.93); 0.958
Scale S (T)	−1.05	(1.78)	0.54	(1.70)	0.25	(1.93)	1.36 (4.18; −6.94 to 9.66); 0.746	2.37 (4.11; −5.78 to 10.52); 0.565	−1.01 (3.94; −8.83 to 6.81); 0.798
Scale avoid	1.11	(1.30)	2.21	(1.25)	−0.08	(1.41)	−1.46 (2.69; −6.79 to 3.87); 0.588	−0.11 (2.64; −5.34 to 5.13); 0.968	−1.35 (2.53; −6.38 to 3.67); 0.594
Disability by PDI^a^	−1.98	(1.59)	−3.95	(1.53)	−1.45	(1.72)	−5.30 (3.64; −12.50 to 1.91) 0.148	−3.08 (3.57; −10.16 to 3.99); 0.390	−2.21; (3.42; −9.00 to 4.58); 0.519
Disability by HIT-6^a^	−0.59	(0.75)	−3.21	(0.72)	−1.23	(0.81)	−0.18 (1.33; −2.82 to 2.47); 0.894	−1.11 (1.31; −3.71 to 1.49); 0.398	0.93 (1.26; −1.56 to 3.42); 0.459
Pain acceptance (CPAQ)^b^	5.34	(1.87)	5.75	(1.80)	4.36	(2.03)	−5.66 (4.43; −14.45 to 3.13); 0.204	−7.98 (4.35; −16.61 to 0.65);0.070	2.32 (4.18; −5.96 to 10.60); 0.580

*Change from pre to post is based on estimated marginal means (EMM). Mean difference is based on EMM at post-measure. Analyses using linear mixed models for repeated measures, including group, time, and the group x time interaction as fixed factors, and a random intercept to model interindividual differences, based on diagonal covariance matrices and restricted maximum likelihood estimation. Headache days, Headache index, and Medication days each refer to a 28-day period. The HTSAQ comprises 26 triggers. Since two triggers are not listed before, and two triggers (i.e., smoking, menstrual cycle) do not apply to everyone, only the data of 22 triggers was taken into account. miCBT, migraine-specific, integrative cognitive-behavioral therapy program; RLX, relaxation training; WLC, waiting-list control-group; SE, standard error; HDI, Headache Disability Index; DASS, Depression, Anxiety and Stress Scales, total score; HMSE-G-SF, Headache Management Self-Efficacy Scale, German version, Short-Form; HTSAQ, Headache Triggers Sensitivity and Avoidance Questionnaire; S (O), Sensitivity compared with Others; S (T), Sensitivity compared with Time of least sensitivity; PDI, Pain Disability Index; HIT-6, Headache Impact Test; CPAQ, Chronic Pain Acceptance Questionnaire.*

a *Higher values mirror higher burden or higher avoidance.*

b *Higher values mirror higher self-efficacy or higher acceptance*.

**Figure 3:**
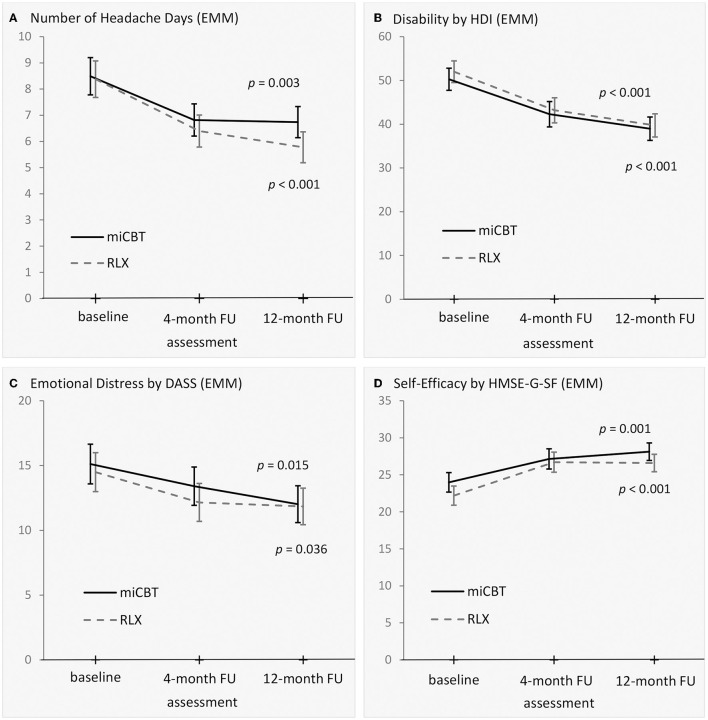
Primary outcomes (by intention-to-treat, *N* = 104) of the follow-up analyses. **(A)** Headache days each refer to a 28-day period. **(B)** HDI and **(C)** DASS: Higher values mirror higher burden. **(D)** HMSE-G-SF: higher values mirror higher self-efficacy. Values are based on estimated marginal means (EMM). The error bars present the standard error. Assessment is at baseline, 4-months post-treatment, and 12-months post-treatment. Analyses used a linear mixed model for repeated measures. The *p*-value each refers to a within-group comparison from baseline to 12-month follow-up (miCBT: upper *p*-value; RLX: lower *p*-value). Since two participants of the waiting-list control group were not allocated to one of the two treatment conditions for the follow-up, *N* = 104 (Figure 2). miCBT, migraine-specific, integrative cognitive-behavioral therapy program; RLX, relaxation training; EMM, estimated marginal means; FU, follow-up; HDI, Headache Disability Index; DASS, Depression, Anxiety and Stress Scales, total score; HMSE-G-SF, Headache Management Self-Efficacy Scale, German version, Short-Form.

Analyses of long-term effects (miCBT vs. RLX; Figure 3; Supplementary Tables 5, 6) did not reveal any significant group differences between the two treatments for changes from baseline to the 4- and 12-month follow-up for primary and secondary outcomes (all *p*-values > 0.05). However, the within-group change from baseline to 12-month follow-up showed significant improvements in all primary and most of the secondary outcomes. Here, the frequency of headache days decreased by 1.75 (miCBT) or 2.60 (RLX) days from baseline to 12-month follow-up (each based on a 28-day period, difference based on estimated marginal means, Figure 3, Supplementary Table 5). The within-group effect sizes (Cohen's *d*_av_) of the pre to 12-month follow-up changes ranged from 0.04 (HTSAQ, Scale B) to 0.80 (CPAQ-D; Supplementary Table 7).

After 12-months, a large proportion of the participants reported to continue practicing some form of relaxation (81% miCBT; 78% RLX; *p* = 1), whereas the participants of the RLX-group reported a higher extent of weekly relaxation praxis in minutes, which however did not differ significantly (CBT: *M* = 40.4; SD = 43.6; RLX: *M* = 59.9; SD = 69.4; *p* = 0.151).

### Response

The responder analyses yielded a similar pattern with no statistically significant difference between the two treatments in the frequency of reliable change (both at 4-month and 12-month follow-up; Supplementary Table 8). For the symptom-related outcomes (headache days, headache index, and medication days), the response rate was higher at 12-month follow-up compared to 4-month follow-up (in both treatments). The miCBT, as well as the RLX, showed a ≥30% reduction of headache days for about 44% of the participants at 12-month follow-up.

### Adverse Events and Session Evaluation

Nine participants (miCBT: four; RLX: five) reported a total of 13 adverse events on the session evaluation form. All reported events were classified as temporary and not serious. Five participants (miCBT: four; RLX: one) discontinued the treatment. None of them reported adverse events on the evaluation form. Four of them stated, that the treatment did not meet their expectations. In both treatments, the session evaluation by the GTS-P showed consistently positive assessments (all means >3; Table 3). However, the RLX treatment was rated significantly better in some items and in total (Table 3).

**Table 3:** Session evaluation by the GTS-P.

	**miCBT *n* = 51**		**RLX *n* = 49**		**Mean difference (SE; 95% CI); *p*-value**
Item 1. I was engaged during today's session.	3.65	(0.38)	3.76	(0.39)	−0.11 (0.08; −0.26 to 0.04); 0.147
Item 2. I actively participated in today's session.	3.51	(0.53)	3.74	(0.39)	−0.24 (0.09; −0.42 to −0.05); 0.014
Item 3. I could well comprehend the contents of this session.	3.75	(0.25)	3.85	(0.27)	−0.10 (0.05; −0.20 to >0.00); 0.062
Item 4. Today's session gave me suggestions for coping with my complaints.	3.19	(0.57)	3.50	(0.49)	−0.31 (0.11; −0.52 to −0.10); 0.004
Item 5. Today the group was helpful for me.	3.37	(0.49)	3.56	(0.49)	−0.20 (0.10; −0.39 to <0.00); 0.048
Item 6. Today the atmosphere in the group was good.	3.73	(0.41)	3.86	(0.25)	−0.13 (0.07; −0.27 to 0.01); 0.062
Item 7. Overall, I am satisfied with today's session.	3.68	(0.41)	3.80	(0.31)	−0.13 (0.07; −0.267to 0.02); 0.088
Item 8. After today's session, I think that this approach is promising for coping with my complaints.	3.06	(0.62)	3.41	(0.49)	−0.35 (0.11; −0.57 to −0.13); 0.003
Total (mean of all 8 items)	3.49	(0.35)	3.69	(0.31)	−0.19 (0.07; −0.33 to −0.06); 0.004

*Data are mean (standard deviation) unless otherwise stated. All data are observed values. The mean and mean difference each refers to the average value of all 7 sessions. Data refer to all participants, who entered the treatment (n = 100, Figure 2). The p-value refers to a t-Test for independent samples (miCBT vs. RLX). GTS-P: Eeach item or statement is rated on a 5-point Likert scale (0 = “disagree,” to 4 = “agree”; higher scores reflect a more positive evaluation). miCBT, migraine-specific, integrative cognitive-behavioral therapy program; RLX, relaxation training; SE, standard error; GTS-P, Group Therapy Session Evaluation by Patients*.

## Discussion

Behavioral interventions are among the most important strategies to counteract and prevent migraine attacks. Empirical evidence for the efficacy of different behavioral interventions is sparse though. This randomized controlled trial thus investigated if a migraine-specific CBT is superior to a standard RLX in the prophylactic treatment of migraine and when compared to a waitlist.

Both interventions (miCBT as well as RLX) led to a significantly higher pre-post improvement in self-efficacy compared to the WLC. Statistically summarized as BT, both behavioral interventions led to a significantly higher pre-post reduction in headache activity (assessed by the headache index) compared to the WLC. In the follow-up, both interventions yielded significant within-group improvements in most of the outcomes. However, and contrary to expectations, miCBT was not superior to RLX in improving clinical outcomes, neither in the pre-post analyses nor in the follow-up analyses. There were only significant differences in the evaluation of the treatment by participants, with RLX doing unexpectedly better. The question arises as to why the miCBT, which was more tailored to migraine, was not superior. An explanation for the strength of the RLX could be its focus on a single, easy-to-use technique (the PMR) in contrast to the complex, more demanding miCBT. The participants of the RLX found the relaxation exercises probably easier to implement since CBT techniques are supposed to be more challenging to apply in everyday life (51). In contrast, the miCBT provided a novel intervention in each session, which in some cases could have led to excessive demands on the participants. Increasing the treatment dose in miCBT to more sessions could lead to larger effects. Another explanation for an advantage of the RLX could be that this intervention may have addressed both declarative and non-declarative memory (52). While declarative processing was stimulated by information about relaxation and talking about planned relaxation practice, non-declarative processing was also encouraged through the real practice of relaxation during the session. The miCBT focused more on patient education, the analysis of migraine-associated behavior, and the setting of behavior change goals. In contrast, there was less practical exercise, so that non-declarative processing of the content may have been neglected here. A future migraine-specific CBT program could benefit from even more hands-on exercises during the session (e.g., role-plays, practicing skills). While the group therapy setting offers the possibility of an interpersonal exchange of experiences, the face-to-face setting is probably more suitable for exposure-based interventions in the context of trigger management.

Since RLX represents one of the most evidence-based treatments for migraine prophylaxis, having even an impact on physiological parameters, (17) the non-inferiority of miCBT offers patients a validated treatment option. The next step would be to identify patient characteristics that are associated with greater benefits from one treatment or the other (53).

Beyond this, there is a growing body of literature showing that different psychological interventions (for both migraine and other disorders) lead to similar improvements, (19, 54) which is in line with the present results. While we could not find a superior effect of migraine-specific CBT interventions, other change mechanisms (e.g., an increase in self-efficacy) may be of more decisive importance. At post-measurement, both active treatments (miCBT and RLX) yielded a significant increase in headache-related self-efficacy compared to WLC. In both treatments, significant improvements were observed in most of the outcomes from pre to 4-month as well as to 12-month follow-up, providing support for emerging treatment effects in the long-term. The reduction of about two headache days from baseline to follow-up is comparable to observed improvements in recent randomized clinical trials on non-medical treatments (19, 55). The improvement in self-efficacy already at post-measurement was probably central to the further success of both treatments. This finding is in line with the results of a previous study that observed large increases in headache self-efficacy through behavior therapy and emphasized the high importance of this parameter (56). Probably, patients are capable to select and employ behavioral techniques that are suitable for their individual needs by self-management (51). Since even a low-threshold non-medical intervention (lifestyle adaption) for migraine prophylaxis was able to bring about an improvement in self-efficacy, (57) it is worthwhile to consider making appropriate interventions available to as many patients as possible.

Strengths of the study include the novel miCBT, the three-armed design, including RLX as a strong comparator, and a long follow-up interval. Practicability is supported by the low drop-out rate. Limitations are the monocentric design and the lack of a control group for the follow-up. Since there was no precise assessment of behavioral changes implemented by the participants, a more accurate recording of at least some basic behavior strategies (e.g., monitoring the frequency of relaxation praxis by an online diary) would have been desirable. Further, the sample size of the study can be considered too low for robust interpretation of miCBT vs. RLX differences or a strict test regarding non-inferiority.

Future research should examine if subgroups (i.e., patients with high psychosocial burden, such as a high trigger avoidance or a strong fear of attacks) benefit from a more tailored intervention, i.e., the miCBT. Future studies also could examine the potential effects of higher treatment doses. Methodological improvements, such as the use of a control group for a long-term follow-up or a more accurate assessment of patients' behavioral changes in daily life, should be considered.

In conclusion, a novel, group-based migraine-specific CBT program could be shown to have similar effects on migraine prophylaxis compared to relaxation training. Both treatments were leading to a significant improvement in self-efficacy compared to a WLC. Both treatments also showed the potential to reduce headache activity and headache-related impairment, but there is still no strong evidence of efficacy in this regard, and further studies are needed to evaluate the efficacy of behavioral therapy for migraine prophylaxis.

## Data Availability Statement

The raw data supporting the conclusions of this article will be made available by the corresponding authors upon reasonable request.

## Ethics Statement

The study was approved by the Ethics Committee of the State Chamber of Medicine in Rhineland-Palatinate, Germany, reference number 837.291.16 (10610). The patients/participants provided their written informed consent to participate in this study.

## Author Contributions

TK, CG, EL-L, and MW: study design and conception TK: supervision and training of the research staff and therapists, conception of the miCBT- and the RLX, overall responsibility for day-to-day running of the study, acquisition, and drafting of the manuscript. BB: advice on randomization and programming the sequence for the analysis of the headache diary data. IH: acquisition of the data and conception of the RLX. SH, BB, and TK: analysis and interpretation of the data. SH, IH, MW, and CG: critical revision of the manuscript. MW: advice on all statistical aspects of the trial. All authors contributed to the manuscript and approved the submitted version.

## Funding

This trial was funded by the German Migraine and Headache Society (DMKG, e.V.) and by internal funds (Department of Psychology, Johannes Gutenberg University of Mainz). The publication fee is largely taken over by the University Library of the Johannes Gutenberg University of Mainz.

## Conflict of Interest

CG has received honoraria for consulting and lectures within the past 3 years from Allergan Pharma, Lilly, Novartis Pharma, Hormosan Pharma, Grünenthal, Sanofi-Aventis, Weber & Weber, Lundbeck Perfood, and TEVA and is honorary secretary of the German Migraine and Headache Society. EL-L has received honoraria for lectures within the past 3 years from Allergan Pharma, Lilly, and TEVA. EL-L and TK published a treatment manual about CBT for migraine. The remaining authors declare that the research was conducted in the absence of any commercial or financial relationships that could be construed as a potential conflict of interest.

## Publisher's Note

All claims expressed in this article are solely those of the authors and do not necessarily represent those of their affiliated organizations, or those of the publisher, the editors and the reviewers. Any product that may be evaluated in this article, or claim that may be made by its manufacturer, is not guaranteed or endorsed by the publisher.
