# Supplementary material for: Efficacy of Cognitive-Behavioral Therapy for the Prophylaxis of Migraine in Adults: A Three-Armed Randomized Controlled Trial
Source: Front Neurol. 2022 Apr 28;13:852616. doi: 10.3389/fneur.2022.852616 (PMC9101654; doi:10.3389/fneur.2022.852616)
Supplement: Supplementary file 1 [file Data_Sheet_1.pdf]

Supplementary Material

Supplementary Table 1. Primary and secondary efficacy outcomes (by completer, $N = 97$) of the pre-post sensitivity mixed model analyses

	Between-group differences<br>Mean difference at post (SE; 95% CI); *p*-value		
	miCBT vs WLC	RLX vs WLC	miCBT vs RLX
Primary Outcomes			
Headache days	0.21 (1.17; –2.11 to 2.53); 0.858	–0.84 (1.14; –3.10 to 1.42); 0.461	1.05 (1.11; –1.15 to 3.25); 0.345
Disability by HDI [a]	0.96 (5.04; –9.04 to 10.95); 0.850	5.37 (4.90; –4.36 to 15.10); 0.276	–4.42 (4.77; –13.89 to 5.06); 0.357
Emotional distress (DASS) [a]	2.53 (2.68; –2.79 to 7.84); 0.347	2.64 (2.60; –2.53 to 7.81);0.314	–0.11 (2.54; –5.14 to 4.92); 0.965
Self-efficacy (HMSE-G-SF) [b]	5.22 (2.13; 0.99 to 9.44); 0.016	4.49 (2.07; 0.38 to 8.61); 0.033	0.72 (2.02; –3.28 to 4.73); 0.721
Secondary Outcomes			
Headache index	0.01 (0.04; –0.08 to 0.10); 0.865	–0.05 (0.04; –0.13 to 0.04); 0.304	0.05 (0.04; –0.03 to 0.14); 0.218
Medication days	–0.24 (1.03; –2.29 to 1.81); 0.816	–0.59 (1.00; –2.58 to 1.40); 0.557	0.35 (0.98; –1.59 to 2.29); 0.721
Triggers (HTSAQ) [a]			
- Scale Triggers	0.79 (3.09; –5.35 to 6.92); 0.799	0.35 (3.01; –5.62 to 6.32); 0.908	0.44 (2.93; –5.37 to 6.25); 0.881
- Scale S (O)	–0.31 (3.67; –7.59 to 6.97); 0.934	1.60 (3.57; –5.49 to 8.68); 0.656	–1.90 (3.47; –8.80 to 5.00); 0.585
- Scale S (T)	0.64 (4.29; –7.87 to 9.15); 0.882	2.76 (4.17; –5.52 to 11.05); 0.510	–2.12 (4.06; –10.19 to 5.95); 0.603
- Scale Avoid	–2.29 (2.72; –7.68 to 3.11); 0.402	0.19 (2.64; –5.06 to 5.44); 0.942	–2.48 (2.57; –7.59 to 2.63); 0.338
Disability by PDI [a]	–5.89 (3.85; –13.54 to 1.75); 0.129	–3.26 (3.75; –10.71 to 4.18); 0.386	–2.63 (3.65; –9.88 to 4.62); 0.473
Disability by HIT-6 [a]	–0.39 (1.37; –3.11 to 2.34); 0.778	–1.28 (1.34; –3.93 to 1.37); 0.341	0.89 (1.30; –1.69 to 3.47); 0.496
Pain Acceptance (CPAQ) [b]	–5.51 (4.59; –14.62 to 3.61); 0.233	–7.86 (4.47; –16.73 to 1.01); 0.082	2.35 (4.35; –6.29 to 10.99); 0.590

Note. Mean difference is based on estimated marginal means (EMM) at post-measure. Analyses used a linear mixed model for repeated measures, including group, time, and the group x time interaction as fixed factors, and a random intercept to model interindividual differences, based on diagonal covariance matrices and restricted maximum likelihood estimation. Headache days, Headache index, and Medication days each refer to a 28-day period. The HTSAQ comprises 26 triggers. Since two triggers are not listed before, and two triggers (ie, smoking, menstrual cycle) do not apply to everyone, only the data of 22 triggers was taken into account. miCBT, migraine-specific, integrative cognitive-behavioral therapy program; RLX, relaxation training; WLC, waiting-list control-group; SE, standard error; HDI, Headache Disability Index; DASS, Depression, Anxiety and Stress Scales, total score; HMSE-G-SF, Headache Management Self-Efficacy Scale, German version, Short-Form; HTSAQ, Headache Triggers Sensitivity and Avoidance Questionnaire; S (O), Sensitivity compared with Others; S (T), Sensitivity compared with Time of least sensitivity; PDI, Pain Disability Index; HIT-6, Headache Impact Test; CPAQ, Chronic Pain Acceptance Questionnaire.

[a] Higher values mirror higher burden or higher avoidance.
[b] Higher values mirror higher self-efficacy or higher acceptance.

Supplementary Table 2. Primary and secondary efficacy outcomes (by completer, $N = 97$) of the pre-post sensitivity analyses by analysis of covariance

	Between-group differences<br>Mean difference at post (SE; 95% CI); *p*-value		
	miCBT vs WLC	RLX vs WLC	miCBT vs RLX
Primary Outcomes			
Headache days	–0.53 (0.91; –2.34 to 1.28); 0.564	–1.41 (0.89; –3.17 to 0.35); 0.116	0.88 (0.86; –0.83 to 2.59); 0.310
Disability by HDI [a]	1.12 (2.55; –3.94 to 6.17); 0.663	3.19 (2.48; –1.74 to 8.12); 0.202	–2.07 (2.42; –6.88 to 2.73); 0.394
Emotional distress (DASS) [a]	–0.77 (1.86; –4.47 to 2.93); 0.680	–0.79 (1.82; –4.39 to 2.82); 0.666	0.02 (1.74; –3.44 to 3.47); 0.993
Self-efficacy (HMSE-G-SF) [b]	4.26 (1.82; 0.65 to 7.87); 0.021	4.92 (1.76; 1.42 to 8.42); 0.006	–0.66 (1.73; –4.10 to 2.77); 0.703
Secondary Outcomes			
Headache index	–0.03 (0.03; –0.09 to 0.03); 0.316	–0.07 (0.03; –0.13 to –0.01); 0.015	0.04 (0.03; –0.01 to 0.10); 0.143
Medication days	–0.72 (0.84; –2.39 to 0.95); 0.395	–0.89 (0.82; –2.51 to 0.73); 0.276	0.18 (0.79; –1.40 to 1.75); 0.825
Triggers (HTSAQ) [a]			
- Scale Triggers	1.88 (1.85; –1.79 to 5.54); 0.312	0.45 (1.79; –3.12 to 4.01); 0.804	1.43 (1.75; –2.04 to 4.91); 0.415
- Scale S (O)	1.62 (2.22; –2.79 to 6.03); 0.468	0.48 (2.16; –3.81 to 4.77); 0.825	1.14 (2.11; –3.06 to 5.34); 0.591
- Scale S (T)	–1.32 (2.69; –6.66 to 4.01); 0.624	0.48 (2.62; –4.72 to 5.68); 0.854	–1.81 (2.54; –6.86 to 3.24); 0.479
- Scale Avoid	0.11 (1.87; –3.61 to 3.83); 0.954	1.74 (1.81; –1.87 to 5.34); 0.341	–1.63 (1.76; –5.13 to 1.87); 0.358
Disability by PDI [a]	–1.22 (2.40; –5.99 to 3.54); 0.612	–2.61 (2.31; –7.19 to 1.97); 0.261	1.39 (2.27; –3.12 to 5.89); 0.542
Disability by HIT-6 [a]	0.37 (1.09; –1.80 to 2.55); 0.733	–1.93 (1.06; –4.04 to 0.18); 0.072	2.31 (1.05; 0.22 to 4.39); 0.030
Pain Acceptance (CPAQ) [b]	0.07 (2.75; –5.38 to 5.52); 0.981	–0.14 (2.70; –5.51 to 5.23); 0.959	0.21 (2.58; –4.91 to 5.32); 0.936

Note. Mean differences are based on estimated marginal means (EMM) at post-measure. Headache days, Headache index, and Medication days each refer to a 28-day period. The HTSAQ comprises 26 triggers. Since two triggers are not listed before, and two triggers (ie, smoking, menstrual cycle) do not apply to everyone, only the data of 22 triggers was taken into account. miCBT, migraine-specific, integrative cognitive-behavioral therapy program; RLX, relaxation training; WLC, waiting-list control-group; SE, standard error; HDI, Headache Disability Index; DASS, Depression, Anxiety and Stress Scales, total score; HMSE-G-SF, Headache Management Self-Efficacy Scale, German version, Short-Form; HTSAQ, Headache Triggers Sensitivity and Avoidance Questionnaire; S (O), Sensitivity compared with Others; S (T), Sensitivity compared with Time of least sensitivity; PDI, Pain Disability Index; HIT-6, Headache Impact Test; CPAQ, Chronic Pain Acceptance Questionnaire.

[a] Higher values mirror higher burden or higher avoidance.
[b] Higher values mirror higher self-efficacy or higher acceptance.

Supplementary Table 3. Primary and secondary outcomes (by completer) of the pre-post analyses (observed values)

	miCBT				RLX				WLC			
	n = 32				*n* = 36				*n* = 29			
	pre		post		pre		post		pre		post	
Primary Outcomes												
Headache days	8.5	(5.1)	8.0	(4.7)	8.3	(4.1)	6.9	(4.2)	7.4	(3.1)	7.8	(4.8)
Disability by HDI [a]	49.6	(18.4)	44.8	(19.2)	52.1	(16.5)	49.2	(18.8)	49.8	(19.3)	43.8	(21.2)
Emotional distress (DASS) [a]	15.7	(11.6)	13.3	(10.3)	15.8	(10.8)	13.4	(11.0)	11.0	(9.5)	10.7	(9.9)
Self-efficacy (HMSE-G-SF) [b]	24.6	(9.3)	30.3	(5.9)	21.6	(9.5)	29.5	(8.5)	22.5	(10.0)	25.0	(10.2)
Secondary Outcomes												
Headache index [c]	0.26	(0.20)	0.23	(0.20)	0.24	(0.19)	0.18	(0.15)	0.21	(0.12)	0.23	(0.18)
Medication days	6.2	(4.5)	5.7	(4.0)	5.9	(3.2)	5.3	(4.0)	5.4	(3.1)	5.9	(4.1)
Triggers (HTSAQ) [a]												
- Scale Triggers	50.9	(10.2)	52.7	(10.3)	52.1	(12.1)	52.3	(13.2)	52.2	(11.4)	51.9	(12.3)
- Scale S (O)	47.9	(12.4)	49.6	(12.5)	51.3	(13.5)	51.5	(16.5)	50.1	(11.9)	49.9	(13.1)
- Scale S (T)	45.4	(13.8)	44.2	(14.9)	45.7	(16.7)	46.3	(19.9)	43.2	(12.0)	43.5	(14.0)
- Scale Avoid	49.5	(9.4)	50.4	(8.8)	50.7	(10.0)	52.9	(11.4)	52.7	(11.7)	52.7	(11.4)
Disability by PDI [a]	25.2	(10.9)	23.3	(11.9)	29.9	(14.9)	25.9	(15.1)	30.7	(16.0)	29.2	(17.7)
Disability by HIT-6 [a]	59.3	(4.7)	58.8	(4.0)	61.2	(4.7)	57.9	(6.6)	60.3	(3.8)	59.1	(4.9)
Pain Acceptance (CPAQ) [b]	62.5	(16.3)	67.9	(17.6)	59.9	(15.7)	65.6	(16.3)	69.1	(19.9)	73.4	(20.1)

Note. Data are mean (standard deviation), each observed values. Headache days, Headache index, and Medication days each refer to a 28-day period. The HTSAQ comprises 26 triggers. Since two triggers are not listed before, and two triggers (ie, smoking, menstrual cycle) do not apply to everyone, only the data of 22 triggers was taken into account. miCBT, migraine-specific, integrative cognitive-behavioral therapy program; RLX, relaxation training; WLC, waiting-list control-group; SE, standard error; HDI, Headache Disability Index; DASS, Depression, Anxiety and Stress Scales, total score; HMSE-G-SF, Headache Management Self-Efficacy Scale, German version, Short-Form; HTSAQ, Headache Triggers Sensitivity and Avoidance Questionnaire; S (O), Sensitivity compared with Others; S (T), Sensitivity compared with Time of least sensitivity; PDI, Pain Disability Index; HIT-6, Headache Impact Test; CPAQ, Chronic Pain Acceptance Questionnaire.

[a] Higher values mirror higher burden or higher avoidance.
[b] Higher values mirror higher self-efficacy or higher acceptance.
[c] Since values are <1, two decimal places are given.

Supplementary Table 4. Primary and secondary efficacy outcomes (by completer, *N* = 97, pre- to post-assessment) of post-hoc composite analyses (i.e., behavioral therapy group vs waiting-list control group) by analysis of covariance

	Between-group differences<br>Mean difference at post (SE; 95% CI); *p*-value
	BT[a] vs WLC
Primary Outcomes	
Headache days	–1.00 (0.79; –2.56 to 0.57); 0.210
Disability by HDI [b]	2.21 (2.20; –2.16 to 6.58); 0.318
Emotional distress (DASS) [b]	–0.78 (1.61; –3.98 to 2.42); 0.630
Self-efficacy (HMSE-G-SF) [c]	4.61 (1.56; 1.51 to 7.71); 0.004
Secondary Outcomes	
Headache index	–.05 (0.03; –0.10 to < 0.00); 0.047
Medication days	–0.81 (0.72; –2.25 to 0.62); 0.264
Triggers (HTSAQ) [b]	
- Scale Triggers	1.12 (1.59; –2.04 to 4.28); 0.484
- Scale S (O)	1.01 (1.91; –2.78 to 4.81); 0.597
- Scale S (T)	–0.37 (2.32; –4.98 to 4.24); 0.874
- Scale Avoid	0.98 (1.62; –2.23 to 4.19); 0.546
Disability by PDI [b]	–1.97 (2.05; –6.05 to 2.10); 0.339
Disability by HIT-6 [b]	–0.85 (0.96; –2.75 to 1.06); 0.380
Pain Acceptance (CPAQ) [c]	–0.04 (2.39; –4.78 to 4.70); 0.987

Note. Mean differences are based on estimated marginal means (EMM) at post-measure. Headache days, Headache index, and Medication days each refer to a 28-day period. The HTSAQ comprises 26 triggers. Since two triggers are not listed before, and two triggers (ie, smoking, menstrual cycle) do not apply to everyone, only the data of 22 triggers was taken into account. BT, behavioral therapy; WLC, waiting-list control-group; SE, standard error; HDI, Headache Disability Index; DASS, Depression, Anxiety and Stress Scales, total score; HMSE-G-SF, Headache Management Self-Efficacy Scale, German version, Short-Form; HTSAQ, Headache Triggers Sensitivity and Avoidance Questionnaire; S (O), Sensitivity compared with Others; S (T), Sensitivity compared with Time of least sensitivity; PDI, Pain Disability Index; HIT-6, Headache Impact Test; CPAQ, Chronic Pain Acceptance Questionnaire.

[a] This group is including participants, who completed the migraine-specific cognitive-behavioral therapy program or the relaxation training between pre- and post-assessment
[b] Higher values mirror higher burden or higher avoidance.
[c] Higher values mirror higher self-efficacy or higher acceptance.

Supplementary Table 45. Primary and secondary outcomes (by intention-to-treat, N = 104) of the follow-up analyses

	Within-group differences				Between-group differences (miCBT vs RLX)	
	miCBT (change from pre to after 4 months) Mean difference (SE); *p*-value	miCBT (change from pre to after 12 months) Mean difference (SE); *p-value*	RLX (change from pre to after 4 months) Mean difference (SE); *p*-value	RLX (change from pre to after 12 months) Mean difference (SE); *p*-value	At 4-month follow-up Mean difference (SE; 95%-CI); *p*-value	At 12-month follow-up Mean difference (SE; 95%-CI); *p*-value
Primary outcomes						
Headache days	–1.67 (0.62); 0.009	–1.75 (0.59); 0.003	–1.98 (0.62); 0.002	–2.60 (0.58); < 0.001	0.42 (0.87; –1.30 to 2.14); 0.628	0.96 (0.84; –0.70 to 2.62); 0.254
Disability by HDI [a]	–7.99 (1.74); < 0.001	–11.30 (2.06); < 0.001	–8.82 (1.74); < 0.001	–12.27 (2.06); < 0.001	–0.89 (4.08; –8.98 to 7.20); 0.828	–0.75 (3.76; –8.21 to 6.71); 0.842
Emotional distress (DASS) [a]	–1.71 (1.14); 0.137	–3.10 (1.25); 0.015	–2.34 (1.14); 0.042	–2.65 (1.24); 0.036	1.25 (2.08; –2.87 to 5.38); 0.548	0.17 (2.00; –3.80 to 4.15); 0.931
Self-efficacy (HMSE-G-SF) [b]	3.17 (1.24); 0.012	4.15 (1.19); 0.001	4.51 (1.23); < 0.001	4.41 (1.18); < 0.001	0.44 (1.92; –3.37 to 4.26); 0.818	1.53 (1.67; –1.78 to 4.84); 0.361
Secondary outcomes						
Headache index	–0.03 (0.02); 0.138	–0.03 (0.02); 0.143	–0.06 (0.02); 0.002	–0.09 (0.02); < 0.001	0.04 (0.04; –0.04 to 0.12); 0.353	0.07 (0.04; –0.01 to 0.14); 0.072
Medication days	–1.21 (0.49); 0.015	–1.26 (0.52); 0.017	–1.17 (0.49); 0.018	–1.50 (0.51); 0.004	0.27 (0.66; –1.04 to 1.57); 0.686	0.56 (0.72; –0.87 to 1.99); 0.441
Triggers (HTSAQ) [a]						
- Scale Triggers	1.51 (0.96); 0.119	0.75 (0.92); 0.418	–0.68 (0.96); 0.477	–1.34 (0.92); 0.148	2.43 (2.30; –2.13 to 6.99); 0.292	2.33 (2.34; –2.30 to 6.96); 0.321
- Scale S (O)	2.41 (1.29); 0.065	1.66 (1.32); 0.210	–0.83 (1.28); 0.521	–0.62 (1.31); 0.637	2.95 (2.73; –2.45 to 8.35); 0.281	2.00 (2.77; –3.48 to 7.47); 0.472
- Scale S (T)	–0.93 (1.47); 0.526	–2.02 (1.81); 0.266	–1.62 (1.47); 0.272	–1.44 (1.80); 0.428	0.16 (3.23; –6.25 to 6.57); 0.961	–1.11 (3.25; –7.56 to 5.34); 0.734
- Scale Avoid	0.73 (1.12); 0.513	1.92 (1.00); 0.058	–1.16 (1.11); 0.300	–0.75 (1.00); 0.453	1.25 (2.07; –2.87 to 5.36); 0.550	2.02 (2.10; –2.13 to 6.17); 0.336
Disability by PDI [a]	–4.90 (1.49); 0.001	–8.46 (1.63); < 0.001	–4.44 (1.48); 0.003	–5.91 (1.63); < 0.001	–3.20 (2.78; –8.72 to 2.31); 0.252	–5.29 (2.79; –10.81 to 0.24); 0.060
Disability by HIT-6 [a]	–1.84 (0.74); 0.014	–3.73 (0.78); < 0.001	–3.03 (0.73); < 0.001	–3.86 (0.78); < 0.001	0.14 (1.22; –2.28 to 2.56); 0.911	–0.93 (1.15; –3.21 to 1.34); 0.417
Pain Acceptance (CPAQ) [b]	8.09 (1.81); < 0.001	9.27 (1.92); < 0.001	7.04 (1.81); < 0.001	12.48 (1.91); < 0.001	3.29 (3.33; –3.33 to 9.90); 0.327	–0.98 (3.47; –7.84 to 5.89); 0.779

Note. Mean differences are based on estimated marginal means (EMM). Analyses used a linear mixed model for repeated measures, including group, time, and the group x time interaction as fixed factors, and a random intercept to model interindividual differences, based on an auto-regressive structure with heterogenous variances (ARH1) and restricted maximum likelihood estimation. Since two participants of the waiting-list control group were not allocated to one of the two treatment conditions for the follow-up, N = 104 (Figure 1). Headache days, Headache index, and Medication days each refer to a 28-day period. The HTSAQ comprises 26 triggers. Since two triggers are not listed before, and two triggers (ie, smoking, menstrual cycle) do not apply to everyone, only the data of 22 triggers was taken into account. miCBT, migraine-specific, integrative cognitive-behavioral therapy program; RLX, relaxation training; WLC, waiting-list control-group; SE, standard error; HDI, Headache Disability Index; DASS, Depression, Anxiety and Stress Scales, total score; HMSE-G-SF, Headache Management Self-Efficacy Scale, German version, Short-Form; HTSAQ, Headache Triggers Sensitivity and Avoidance Questionnaire; S (O), Sensitivity compared with Others; S (T), Sensitivity compared with Time of least sensitivity; PDI, Pain Disability Index; HIT-6, Headache Impact Test; CPAQ, Chronic Pain Acceptance Questionnaire.

[a] Higher values mirror higher burden or higher avoidance.
~~‡~~[b] Higher values mirror higher self-efficacy or higher acceptance.

Supplementary Table ~~5~~6. Primary and secondary outcomes (by completer) of the follow-up analyses (observed values)

	miCBT						RLX					
	Pre *n* = 45		4-month FU *n* = 44		12-month FU *n* = 36		Pre *n* = 46		4-month FU *n* = 44		12-month FU *n* = 41	
Primary outcomes												
Headache days	8.1	(4.6)	6.8	(4.1)	5.9	(3.8)	8.1	(4.1)	6.4	(4.6)	5.5	(3.5)
Disability by HDI [a]	49.9	(18.1)	42.2	(20.6)	36.3	(15.5)	53.2	(17.2)	43.9	(20.5)	39.7	(21.0)
Emotional distress (DASS) [a]	14.6	(11.5)	13.1	(11.1)	11.5	(10.2)	14.8	(10.4)	12.1	(9.7)	11.5	(9.1)
Self-efficacy (HMSE-G-SF) [b]	23.7	(9.7)	27.1	(8.4)	27.7	(8.8)	21.9	(9.8)	26.2	(10.3)	26.4	(7.8)
Secondary outcomes												
Headache index [c]	0.24	(0.18)	0.22	(0.21)	0.17	(0.14)	0.24	(0.18)	0.17	(0.15)	0.14	(0.12)
Medication days	6.0	(4.1)	5.0	(3.1)	4.6	(3.5)	5.7	(3.3)	4.8	(3.2)	4.1	(3.1)
Triggers												
(HTSAQ) [a]												
- Scale Triggers	51.3	(9.8)	53.2	(9.9)	51.3	(9.5)	52.3	(12.8)	51.9	(13.4)	50.9	(13.5)
- Scale S (O)	48.9	(11.9)	51.9	(12.4)	49.5	(12.7)	51.0	(13.8)	50.3	(15.5)	50.0	(15.6)
- Scale S (T)	44.0	(12.9)	43.4	(13.7)	41.1	(15.0)	46.0	(16.3)	44.5	(18.4)	44.0	(17.6)
- Scale Avoid	50.3	(10.0)	51.3	(9.0)	50.7	(7.9)	51.6	(10.8)	50.9	(11.5)	50.3	(11.5)
Disability by PDI [a]	27.2	(11.9)	22.4	(11.8)	17.9	(10.7)	30.6	(15.1)	25.8	(15.8)	24.2	(17.1)
Disability by HIT-6 [a]	59.8	(4.3)	58.3	(4.8)	56.2	(4.4)	61.2	(4.5)	58.2	(6.4)	57.5	(6.8)
Pain Acceptance (CPAQ) [b]	63.8	(17.4)	71.6	(18.0)	74.8	(17.2)	61.1	(16.6)	68.2	(15.0)	74.7	(16.0)

Note. Data are mean (SD). Headache days, Headache index, and Medication days each refer to a 28-day period. The HTSAQ comprises 26 triggers. Since two triggers are not listed before, and two triggers (ie, smoking, menstrual cycle) do not apply to everyone, only the data of 22 triggers was taken into account. FU, follow-up; miCBT, migraine-specific, integrative cognitive-behavioral therapy program; RLX, relaxation training; WLC, waiting-list control-group; SE, standard error; HDI, Headache Disability Index; DASS, Depression, Anxiety and Stress Scales, total score; HMSE-G-SF, Headache Management Self-Efficacy Scale, German version, Short-Form; HTSAQ, Headache Triggers Sensitivity and Avoidance Questionnaire; S (O), Sensitivity compared with Others; S (T), Sensitivity compared with Time of least sensitivity; PDI, Pain Disability Index; HIT-6, Headache Impact Test; CPAQ, Chronic Pain Acceptance Questionnaire.

[a] Higher values mirror higher burden or higher avoidance.
[b] Higher values mirror higher self-efficacy or higher acceptance.
[c] Since all values are <1, two decimal places are given.

Supplementary Table 67. Within-group effect sizes (by completer) of the 12-month follow-up analyses (based on observed values)

	miCBT<br>n = 36		RLX<br>n = 41	
	Mean difference	d_{av}	Mean difference	d_{av}
Primary Outcomes				
Headache days	–1.5	–0.41	–2.1	–0.59
Disability by HDI [a]	–11.1	–0.69	–12.3	–0.64
Emotional distress (DASS) [a]	–2.2	–0.21	–2.3	–0.23
Self–efficacy (HMSE–G–SF) [b]	4.7	0.50	4.4	0.50
Secondary Outcomes				
Headache index [c]	–0.03	–0.22	–0.08	–0.56
Medication days	–1.2	–0.33	–1.1	–0.38
Triggers (HTSAQ) [a]				
- Scale Triggers	0.9	0.09	–1.4	–0.11
- Scale S (O)	1.8	0.15	–0.6	–0.04
- Scale S (T)	–1.0	–0.07	–1.6	–0.09
- Scale Avoid	1.6	0.19	–1.4	–0.12
Disability by PDI [a]	–7.4	–0.72	–5.9	–0.36
Disability by HIT–6 [a]	–3.2	–0.78	–3.9	–0.67
Pain Acceptance (CPAQ) [b]	10.8	0.65	13.1	0.80

Note. d_{av}: Cohen's *d* for correlated samples. Headache days, Headache index, and Medication days each refer to a 28-day period. The HTSAQ comprises 26 triggers. Since two triggers are not listed before, and two triggers (ie, smoking, menstrual cycle) do not apply to everyone, only the data of 22 triggers was taken into account. miCBT, migraine-specific, integrative cognitive-behavioral therapy program; RLX, relaxation training; WLC, waiting-list control-group; SE, standard error; HDI, Headache Disability Index; DASS, Depression, Anxiety and Stress Scales, total score; HMSE-G-SF, Headache Management Self-Efficacy Scale, German version, Short-Form; HTSAQ, Headache Triggers Sensitivity and Avoidance Questionnaire; S (O), Sensitivity compared with Others; S (T), Sensitivity compared with Time of least sensitivity; PDI, Pain Disability Index; HIT-6, Headache Impact Test; CPAQ, Chronic Pain Acceptance Questionnaire.

[a] Higher values mirror higher burden or higher avoidance.
[b] Higher values mirror higher self-efficacy or higher acceptance.
[c] Since values are <1, two decimal places are given.

Supplementary Table ~~7~~8. Response (by completer) of the follow-up analyses (based on observed values)

	4-month follow-up					12-month follow-up				
	miCBT (n = 44)		RLX (n = 44)		*p*-value	miCBT (n = 36)		RLX (n = 41)		*p*-value
Primary outcomes	Deterioration	Improvement	Deterioration	Improvement		Deterioration	Improvement	Deterioration	Improvement	
Headache days [a]	18.2	40.9	20.5	34.1	0.851	25	44.4	12.2	43.9	0.297
Disability by HDI [b]	2.3	20.5	4.5	34.1	0.358	0	27.8	4.9	34.1	0.414
Emotional distress (DASS) [b]	6.8	15.9	6.8	22.7	0.866	11.1	22.2	14.6	22	0.943
Self-efficacy (HMSE-G-SF) [c]	4.5	20.5	4.5	22.7	1	8.3	25.0	2.4	24.4	0.567
Secondary outcomes										
Headache index [a]	20.5	36.4	22.7	47.7	0.395	27.8	41.7	19.5	58.5	0.313
Medication days [a,d]	22	39.0	14.3	42.9	0.681	20.6	44.1	20.5	48.7	0.952
Triggers (HTSAQ) [b]										
- Scale Triggers	4.5	0	4.5	6.8	0.330	0	0	2.4	7.3	0.243
- Scale S (O)	4.5	2.3	13.6	11.4	0.080	2.8	0	12.2	4.9	0.116
- Scale S (T)	4.5	13.6	11.4	9.1	0.526	11.1	11.1	12.2	7.3	0.918
- Scale Avoid	4.5	2.3	4.5	9.1	0.435	2.8	0	2.4	4.9	0.746
Disability by PDI [b]	2.3	18.2	4.5	27.3	0.481	2.8	27.8	9.8	26.8	0.518
Disability by HIT-6 [b]	2.3	15.9	2.3	25	0.710	0	22.2	2.4	31.7	0.444
Pain Acceptance (CPAQ) [c]	4.5	2.3	4.5	0	1	0	2.8	0	4.9	N/A

Note. Data are % unless otherwise stated. Response in headache days, headache index, and medication days was defined by percentage change from pre to follow-up. Response in psychometric tests (HDI, DASS, HMSE-G-SF, HTSAQ, PDI, HIT-6, CPAQ) was defined by the Reliable Change Index (RCI) from pre to follow-up, applying a 95% range between 1.96 and –1.96. Each *p*-value refers to the differences of frequencies between miCBT and RLX by Fisher's exact test. miCBT, migraine-specific, integrative cognitive-behavioral therapy program; RLX, relaxation training; WLC, waiting-list control-group; SE, standard error; HDI, Headache Disability Index; DASS, Depression, Anxiety and Stress Scales, total score; HMSE-G-SF, Headache Management Self-Efficacy Scale, German version, Short-Form; HTSAQ, Headache Triggers Sensitivity and Avoidance Questionnaire; S (O), Sensitivity compared with Others; S (T), Sensitivity compared with Time of least sensitivity; PDI, Pain Disability Index; HIT-6, Headache Impact Test; CPAQ, Chronic Pain Acceptance Questionnaire.

[a] ≥30% reduction from pre to follow-up was classified as an improvement; ≥30% increase from pre to follow-up was classified as deterioration.
[b] Since higher values mirror higher burden, RCI ≤ –1.97 was defined as improvement, RCI ≥ 1.97 was defined as deterioration.
[c] Since higher values mirror higher self-efficacy or higher acceptance, RCI ≥ 1.97 was defined as improvement, RCI ≤ –1.97 was defined as deterioration.
[d] 5.7% of the sample with a value of zero at pre-measurement was excluded.
